# Cationic nanocarriers induce cell necrosis through impairment of Na^+^/K^+^-ATPase and cause subsequent inflammatory response

**DOI:** 10.1038/cr.2015.9

**Published:** 2015-01-23

**Authors:** Xiawei Wei, Bin Shao, Zhiyao He, Tinghong Ye, Min Luo, Yaxiong Sang, Xiao Liang, Wei Wang, Shuntao Luo, Shengyong Yang, Shuang Zhang, Changyang Gong, Maling Gou, Hongxing Deng, Yinglan Zhao, Hanshuo Yang, Senyi Deng, Chengjian Zhao, Li Yang, Zhiyong Qian, Jiong Li, Xun Sun, Jiahuai Han, Chengyu Jiang, Min Wu, Zhirong Zhang

**Affiliations:** 1 Key Laboratory of Drug Targeting of Ministry of Education, State Key Laboratory of Biotherapy/Collaborative Innovation Center, West China School of Pharmacy, Sichuan University, No. 17, Block 3, Southern Renmin Road, Chengdu, Sichuan 610041, China; 2 State Key Laboratory of Cellular Stress Biology, School of Life Sciences, Xiamen University, Xiamen, Fujian 361005, China; 3 State Key Laboratory of Medical Molecular Biology, Institute of Basic Medical Sciences, Chinese Academy of Medical Sciences, Department of Biochemistry and Molecular Biology, Peking Union Medical College, Beijing 100005, China; 4 Department of Biochemistry and Molecular Biology, School of Medicine and Health Sciences, University of North Dakota, Grand Forks, ND 58202, USA

**Keywords:** cationic nanocarriers, necrosis, Na^+^/K^+^-ATPase, inflammation, damage-associated molecular patterns

## Abstract

Nanocarriers with positive surface charges are known for their toxicity which has limited their clinical applications. The mechanism underlying their toxicity, such as the induction of inflammatory response, remains largely unknown. In the present study we found that injection of cationic nanocarriers, including cationic liposomes, PEI, and chitosan, led to the rapid appearance of necrotic cells. Cell necrosis induced by cationic nanocarriers is dependent on their positive surface charges, but does not require RIP1 and Mlkl. Instead, intracellular Na^+^ overload was found to accompany the cell death. Depletion of Na^+^ in culture medium or pretreatment of cells with the Na^+^/K^+^-ATPase cation-binding site inhibitor ouabain, protected cells from cell necrosis. Moreover, treatment with cationic nanocarriers inhibited Na^+^/K^+^-ATPase activity both *in vitro* and *in vivo*. The computational simulation showed that cationic carriers could interact with cation-binding site of Na^+^/K^+^-ATPase. Mice pretreated with a small dose of ouabain showed improved survival after injection of a lethal dose of cationic nanocarriers. Further analyses suggest that cell necrosis induced by cationic nanocarriers and the resulting leakage of mitochondrial DNA could trigger severe inflammation *in vivo*, which is mediated by a pathway involving TLR9 and MyD88 signaling. Taken together, our results reveal a novel mechanism whereby cationic nanocarriers induce acute cell necrosis through the interaction with Na^+^/K^+^-ATPase, with the subsequent exposure of mitochondrial damage-associated molecular patterns as a key event that mediates the inflammatory responses. Our study has important implications for evaluating the biocompatibility of nanocarriers and designing better and safer ones for drug delivery.

## Introduction

Nanocarrier toxicity raises public health concern^1^. Nanocarriers applied as drug delivery system (DDS) have already been brought into human body^2^. Lipid- or polymer-based nanocarriers are entering the mainstream of delivering both small molecule drugs and macromolecules, which improve their effectiveness or tolerability and simplify their administration^3^. While produced in nano-scale, the nano-sized carriers may take on novel properties and functions, such as offering the ability to interact with complex cellular functions in new ways, which continue to create new biomedical applications^4^. In addition, nanocarriers have diverse potentials in targeted delivery of drug to particular sites by designing the physicochemical properties or surface modification^5^. Surface charge is one of the important characteristics of nanoparticles^6^. Positively-charged nanocarriers, formed by cationic lipids or polymers, are most commonly used in gene delivery as non-viral vectors, including cationic liposomes, polyethylene imines (PEI), chitosan, *etc*.^7,8^. Due to the positively-charged surface, cationic nanocarriers could load and condense nucleic acids simply by electrical interaction with anionic nucleic cargo. Cationic nanocarriers also have other advantages such as simplicity in large-scale production and less stringent vector size limitations while compared with viral vectors^9,10^. Although the benefits brought by nanocarriers in drug delivery have gained much attention and considerable efforts have been devoted to seeking and investigating better cationic carriers, toxicities have always been main barriers in the cationic carrier application^11^.

Potential adverse effects arising from cellular and tissue interactions and immune stimulation of nanocarrier have been reported in publications concerned with preclinical or clinical use of cationic nanocarriers, which are generally associated with the incomplete understanding in the bio-distribution profiles and the immune compatibility of the nanocarriers *in vivo*^12,13,14,15^. Even the most widely used lipoplexes are also confined due to the occurrence of toxicities such as inflammatory toxicity, hepatotoxicity, leukopenia and thrombocytopenia^16,17,18,19^. It is reported that cationic carriers, such as liposomes and PEI, accumulate in lungs to a great extent immediately after administration, which draws attention to pulmonary toxicities of cationic carriers^20,21,22^. Inflammation in lung was observed hours after local administration of lipoplexes (by nebulization or instilled intratracheally) and was characterized by induction of several kinds of proinflammatory cytokines, accumulation of leukocytes and activation of immune cells^23,24,25,26^.

Necrosis, together with apoptosis and autophagic cell death are three types of cell death which have been characterized with their respective morphological changes^27,28^. Necrosis has traditionally been considered as an accidental or passive type of cell death caused by non-physiological stress^29^. However, some evidence recently suggests that the execution of necrotic cell death may be regulated by a set of signal transduction pathways^30,31,32,33^. Necrotic cells are postulated to release endogenous molecules that, using similar nomenclature to pathogen-associated molecular patterns, have been named damage-associated molecular patterns (DAMPs)^34,35^. Of all intracellular components, mitochondria, which have evolved from aerobic prokaryotes according to “endosymbiont hypothesis”, possess many features of their bacterial ancestors, including a circular genome containing CpG DNA and the ability to form N-formyl peptides^36,37^. Thus, mitochondria could function as a major source of DAMPs^38,39^. For instance, mitochondrial DNA (mtDNA) was reported to induce an inflammatory response after injury^37^. The contribution of various DAMPs to inflammation and development of human pathologies has drawn considerable interests in the field of medical science^39^.

It is conceivable that the inflammatory response induced by cationic nanocarriers might be related to cell death and the subsequent release of DAMPs, which in turn chemoattract and activate inflammatory cells. To test this concept, we used cationic nanocarriers including cationic liposomes, PEI-25K and N-trimethyl chitosan as the models to investigate the possible cell death induced by cationic nanocarriers, its mechanism, as well as, how subsequent inflammation occurs.

## Results

### Cationic nanocarriers induce cell necrosis in a positive charge-dependent way

**Figure 1:**
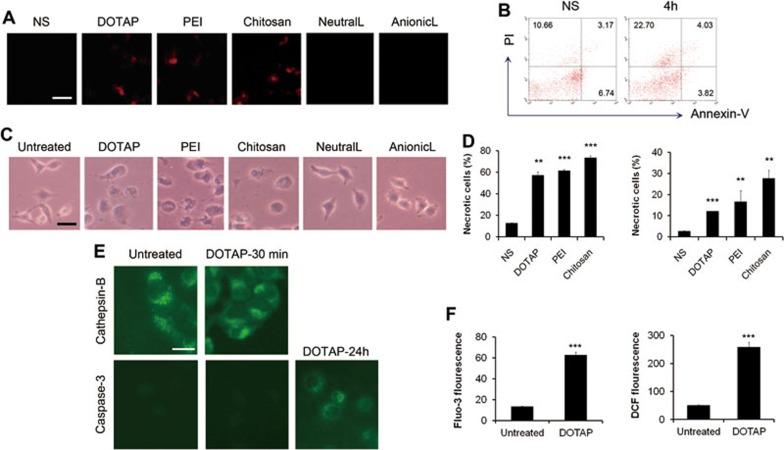
Acute cell necrosis induced by cationic carriers *in vivo* and *in vitro*. **(A)** The detection of propidium iodide (PI)-positive necrotic cells in mouse lungs. Various particles were injected through tail vein of mouse, 2 h later, PI and 4% formaldehyde were perfused through tail vein for the detection of the necrotic cells. **(B)** A representative experiment of the detection of the necrotic cells induced by the injection of cationic liposomes *in vivo* by flow cytometry with Annexin-V and PI staining. C57BL/6 mice were injected with DOTAP liposomes (25 mg/kg). Necrotic cells in BAL fluid were detected 4 h after injection by flow cytometry with Annexin-V and PI staining. *n* = 3/group. **(C)** The morphological change of the cells treated with various nanocarriers *in vitro*. Cells were treated *in vitro* with DOTAP liposome (50 μg/ml), PEI (10 μg/ml), chitosan (50 μg/ml), anionic or neutral liposomes (abbreviated as AnionicL and NeutralL, 50 μg/ml) for 30 min. Cells were subjected to inverted microscope observation. **(D)** The detection of the necrotic cells induced *in vitro* by flow cytometry with Annexin-V and PI staining. Primary lung cells of C57BL/6 mice (left) and A549 cells (right) were treated with cationic carriers for 10 min. Percentages of necrotic cells in PI-positive region are shown. **(E)** A representative experiment of immunofluorescense of Cathepsin-B (green) and Caspase-3. A549 cells were treated with DOTAP liposomes (20 μg/ml) for 30 min. Diffused cytoplasmic cathepsin-B immunoreactivity was evident after the treatment of DOTAP liposome. In contrast, the activation of Caspase-3 was observed after 24 h of treatment. **(F)** A549 cells were treated with DOTAP liposomes, and intracellular Ca^2+^ concentration and ROS levels were detected with Fluo-3/AM and H_2_DCF-DA by flow cytometry, respectively. Data are mean ± SEM; *n* = 3.^**^P< 0.01,^***^P< 0.001 compared with control group by Student's *t*-test.

Since it has been reported that cationic nanocarriers accumulate in lung to a great extent immediately after intravenous injection^20,21,22^, here we focused on how the accumulated cationic particles affect the lung tissue shortly after administration. We investigated whether cationic carriers induce cell death *in vivo*. After the injection of various nanocarriers through mouse tail veins for 2 h, propidium iodide (PI) and 4% formaldehyde were perfused through tail veins subsequently for the detection of necrotic cells within the tissue. Some PI-positive necrotic cells were observed in mouse lungs shortly after injection (Figure 1A). In addition, necrotic cells were also apparently increased in bronchoalveolar lavage (BAL) fluid by flow cytometry with PI and Annexin-V staining (Figure 1B). The PI-positive and Annexin-V-negative region is recognized as a commonly accepted region for necrotic cells and the cells in such a region increased from 10.66% to 22.70% in BAL fluid as detected after 4 h. This indicates that an early lung damage involving cell necrosis was caused by injection of cationic nanocarriers. It has been known that plasmid DNA alone may induce inflammatory response^40^. To investigate the possible mechanisms of the toxicities caused by free cationic nanocarriers, we use the free cationic carriers alone but not plasmids/carriers complexes in most experiments. In addition, the injection of cationic nanocarriers loaded with or without the plasmid in mice both caused pulmonary inflammation and these treatments did not cause a significant difference in mouse survival (Supplementary information, Figure S1).

Next, to exclude the possibility that the inflammatory microenvironment *in vivo* might contribute to cell necrosis, we tested whether cationic nanocarriers induce cell necrosis *in vitro*, using primary cells derived from mouse lungs, human pulmonary epithelial cell line A549 and other cells such as mouse dermal fibroblasts (MDFs), mouse peritoneal macrophages and mouse lymphocytes. The treated cells exhibited the morphological changes of necrotic cells with swelled cytoplasm and ruptured plasma membrane (Figure 1C). The treatment of various cationic particles also led to an apparent increase in the number of necrotic cells as detected by flow cytometry with PI and Annexin-V staining (Figure 1D and Supplementary information, Figure S2). We found that the induction of cell necrosis was in an acute and quick way with the earliest necrosis detected merely after 5 min incubation with cationic particles. Furthermore, diffused cytoplasmic release of cathepsin-B, generally considered a marker of necrosis for the loss of lysosomal membrane integrity, was observed in cells incubated with cationic particles, while untreated cells exhibited dispersed fine granules (Figure 1E). In contrast, apoptotic markers of caspase activation (caspase-3-p20 immunoreactivity) did not appear in cells with or without the incubation of cationic particle at the time when we observed cathepsin-B diffusion, but appeared after 24 h of incubation (Figure 1E). In addition, after 24 h of incubation with cationic nanocarriers, cells were increased in the sub-G1 fraction as detected by flow cytometry and condensation of nuclear chromatin and nuclear fragmentation were found by PI staining (Supplementary information, Figure S3), however, such features of apoptotic cells did not appear in the cells treated with cationic nanocarriers in minutes. Intracellular Ca^2+^ level and reactive oxygen species (ROS) production were also measured, and both showed an increase after the treatment of cationic nanocarriers (Figure 1F).

**Figure 2:**
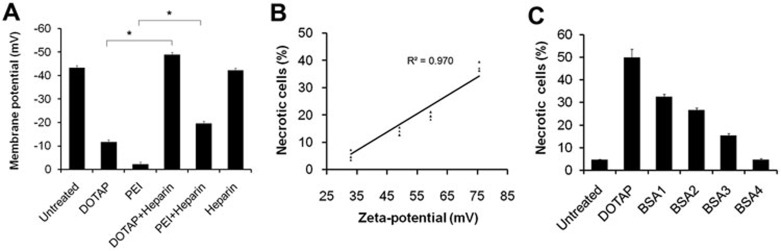
Cell depolarization and cell necrosis triggered by cationic carriers in a positive charge-dependent way. **(A)** A549 cell depolarization caused by cationic particles was determined by diBA-C4-(3) after 1 min of treatment. Heparin (400 μg/ml) was added to neutralize the cationic charge. **(B)** Percentages of necrotic cells after incubation of A549 cells with liposomes of various zeta-potentials. **(C)** Cationic charge was neutralized by BSA with concentrations of 40, 80, 160, 640 μg/ml. Data are mean ± SEM; *n* = 3.^*^P< 0.05 compared with control group by Student's *t*-test.

To further study how the particle surface charge is involved in the interaction of cationic nanocarriers with cells, we determined the change in cell membrane potential with membrane potential-sensitive fluorescent dye bis(1,3-dibutylbarbituric acid) trimethine oxonol (diBA-C4-(3)) by flow cytometry. The addition of cationic particles induced apparent cell depolarization from −43.3 ± 4.4 mV to −11.6 ± 3.2 mV (Figure 2A). The cell depolarization was detected in less than 60 s of treatment, which occurred before the earliest cell necrosis that we have determined. These results suggest that the surface charge of cationic nanocarrier apparently affects the cell membrane potential before cell necrosis. In the next set of the experiment we found that cationic carriers induced cell necrosis in a charge-dependent manner. Cells were treated with cationic carriers of the same concentration, but of different zeta-potentials (Figure 2B). The cytotoxicity of cationic liposome increased with the increase in its zeta-potential. Linear regression analysis showed a good correlation between the zeta-potentials of cationic liposomes and the percentages of induced necrotic cell death (R^2^= 0.970). The number of necrotic cells decreased when the positive charge was neutralized by anionic albumin from bovine serum (BSA; Figure 2C) or anionic heparin. These findings suggest that cationic carriers may induce rapid cell necrosis in a positive charge-dependent way.

### Cell necrosis induced by cationic nanocarriers does not require RIP1 and Mlkl pathway

**Figure 3:**
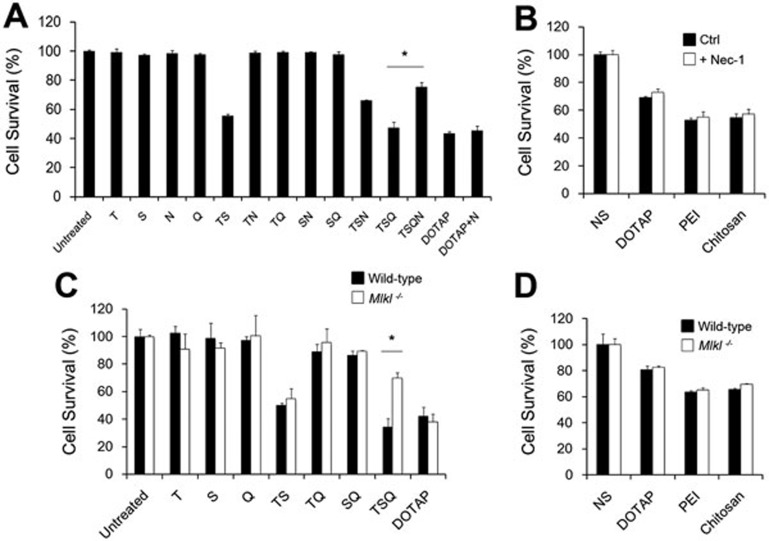
RIP1 and Mlkl might not be involved in cationic nanocarrier-induced cell necrosis. Mouse dermal fibroblasts (MDFs) were isolated from both wild-type and *Mlkl^−/−^* mice. Abbreviations and concentrations are as follows: T, hTNF (100 ng/ml); S, Smac-mimetic (500 nM); N, Necrostatin-1 (50 μM); Q, QVD-OPH (5 μM); DOTAP liposome (25 μg/ml); PEI (5 μg/ml); Chitosan (25 μg/ml). **(A**, **C)** MDFs were treated as indicated for 18 h. **(B**, **D)** MDFs were treated with cationic carriers for 30 min. Cell viability was determined by MTT assay. Data are expressed as mean ± SEM of triplicates.^*^P < 0.05 by Student's *t*-test.

Recently, tumor necrosis factor (TNF)-induced necrosis, necroptosis, was studied and receptor interacting protein 1 (RIP1) and mixed lineage kinase domain-like protein (Mlkl) phosphorylated by RIP3 were both characterized as the crucial downstream components^30,31,32,33^. To find out whether cell necrosis induced by cationic carriers involves RIP1 and Mlkl, we used specific RIP1 inhibitor, Necrostatin-1, and *Mlkl^−/−^* mice to test the cytotoxicity of cationic nanocarriers. However, cells were not protected from cationic carrier-induced necrosis with either inhibition of RIP1 or knockout of Mlkl as compared with controls after 18 h or 30 min of treatment (Figure 3). In contrast, as the positive control, cells treated with necrostatin-1 or *Mlkl^−/−^* cells were resistant to necroptosis induced by the combination of TNF-α (T), Smac-mimetic (S), and the caspase-inhibitor QVD-OPH (Q). Thus, cell necrosis induced by cationic nanocarriers might not involve RIP1- or Mlkl-associated pathways.

### Cell necrosis induced by cationic nanocarriers involves impairment of Na^+^/K^+^-ATPase activity

**Figure 4:**
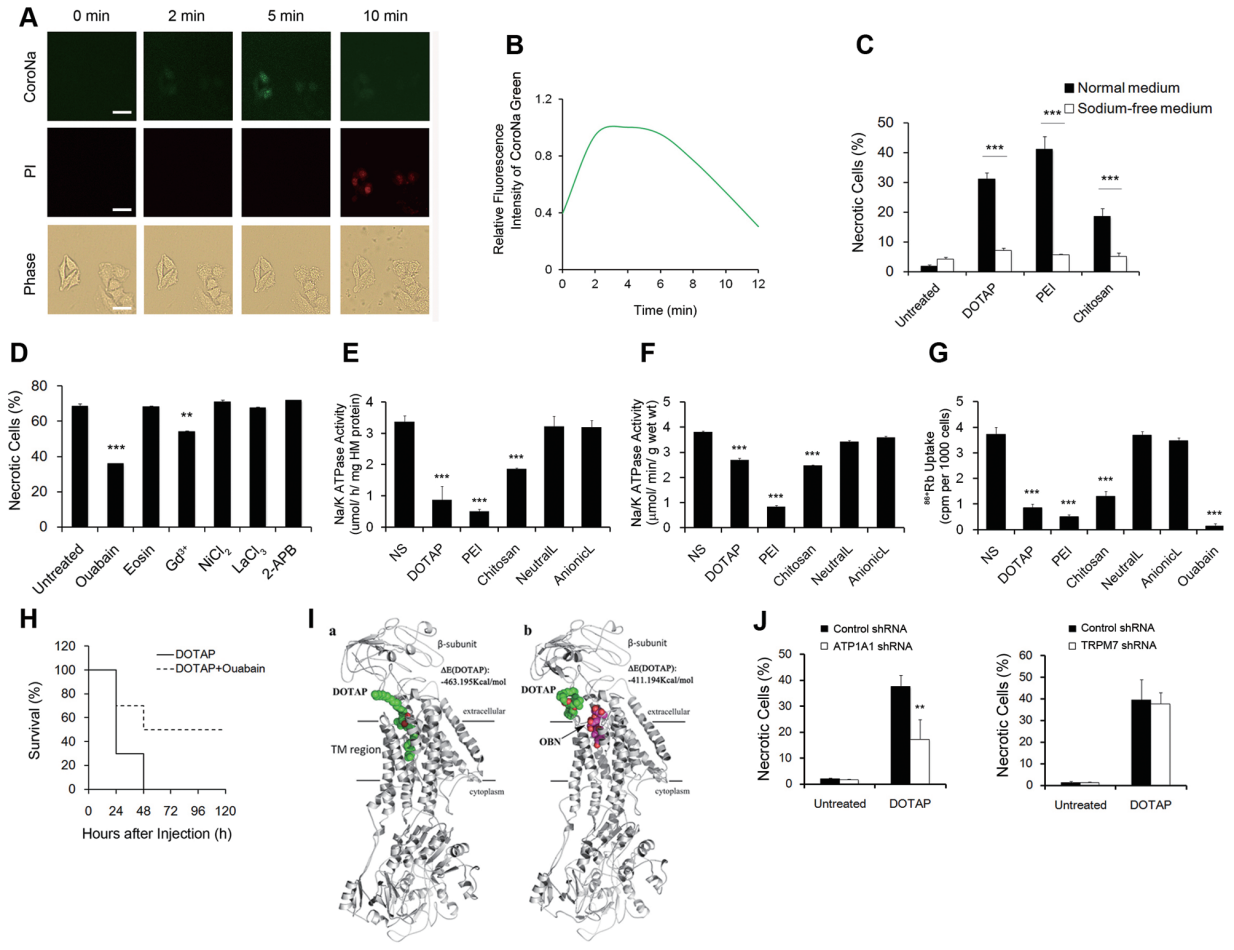
Cell necrosis induced by cationic carriers involves impairment of Na^+^/K^+^-ATPase activity. **(A)** Representative images of A549 cells after the addition of DOTAP liposomes (50 μg/ml). Cells were loaded with fluorescence sodium indicator CoroNa Green and stained with PI. Scale bar, 20 μm. **(B)** The fluorescence intensities of CoroNa Green in one field were recorded and analyzed using ImageJ software. **(C)** A549 cells were cultured in medium with or without sodium for 1 h and then treated with cationic carriers for 10 min. DOTAP liposome (50 μg/ml); PEI (10 μg/ml); Chitosan (10 μg/ml). **(D)** A549 cells were pretreated with inhibitors including ouabain (2 μM), eosin (5 μM), Gd^3+^(20 μM), NiCl_2_ (1 mM), LaCl_3_(0.1 mM) or 2-APB (50 μM) for 30 min and DOTAP liposomes (100 μg/ml) were added. Necrotic cells were detected by flow cytometry with PI staining in 10 min. **(E**, **F)** Na^+^/K^+^-ATPase activity in cultured cells and in tissues treated with cationic carriers. A549 cells were treated with DOTAP liposome (50 μg/ml), PEI (10 μg/ml), Chitosan (50 μg/ml), Neutral liposomes (NeutralL, 50 μg/ml) or Anionic liposomes (AnionicL, 50 μg/ml) for 5 min and heavy-membrane fraction of the cells were used for Na^+^/K^+^-ATPase activity assay **(E)**. Mice were injected with DOTAP liposome (25 mg/kg), PEI (5 mg/kg), Chitosan (25 mg/kg), Neutral liposomes (25 mg/kg) or Anionic liposomes (25 mg/kg) and 20 min later homogenates of lungs were prepared for Na^+^/K^+^-ATPase activity assay **(F)**. **(G)** Uptake of ^86^Rb^+^ in A549 cells treated with cationic nanocarriers. A549 cells were incubated with DOTAP liposome (50 μg/ml), PEI (10 μg/ml), Chitosan (50 μg/ml), Neutral liposomes (50 μg/ml) or Anionic liposomes (50 μg/ml) for 2 min and treatment of 5 μM ouabain was used as positive control. *n* = 3. **(H)** Mice were pretreated with or without ouabain (5 μg/mice) for 10 min and subsequently injected with DOTAP liposomes (100 mg/kg) through tail veins every 24 h for two days and mouse survival were recorded every 24 h, *n* = 10. **(I)** Complex structures were calculated. (a) for Na^+^/K^+^-ATPase-DOTAP and (b) for Na^+^ /K^+^-ATPase-ouabain/DOTAP. **(J)** Control-shRNA, Na^+^/K^+^-ATPase-shRNA (ATP1A1-shRNA) and TRPM7-shRNA transfected A549 cells were treated with DOTAP liposomes (50 μg/ml) for 5 min before analysis by flow cytometry. Data are mean ± SEM; *n* = 3.^**^P< 0.01,^***^P< 0.001 compared with control group by Student's *t*-test.

Considering that cell swelling is often associated with disrupted ionic homeostasis, such as intracellular overload of Na^+^, we monitored ionic homeostasis changes during the interaction with cationic carriers. By staining cells with CoroNa Green, a significant increase in the intracellular concentration of Na^+^ upon the addition of cationic carriers was found (Figure 4A). The CoroNa green fluorescence intensity was recorded by a time-lapse fluorescence microscope (Figure 4B). While the concentration of Na^+^ maintained high for a while, the fluorescence began to drop at the time point when cell membrane became permeable to PI which indicated cell membrane damage. Finally, Na^+^ concentration dropped to the background level as cells were PI-positive. In addition, we cultured cells in normal medium or sodium-free medium before the addition of cationic carriers. Sodium depletion in culture medium significantly inhibited cationic carrier-induced cell necrosis (Figure 4C). These results indicate that Na^+^ overload plays a critical role in induction of cell necrosis by cationic carriers.

Furthermore, to investigate how cationic carriers induced Na^+^ overload in cell, cells were pretreated with several bioactive inhibitors for 30 min, including ouabain (Na^+^/K^+^-ATPase inhibitor, binds to cation-binding site), eosin (Na^+^/K^+^-ATPase inhibitor, binds to ATP-binding site), Gd^3+^(stretch-activated cation channel blocker), NiCl_2_ (the T-type voltage-dependent calcium channel blocker), LaCl_3_ and 2-APB (non-voltage-sensitive calcium channel blockers). An evident protection of cells from necrotic death was achieved by pretreatment of cells with cell membrane Na^+^/K^+^-ATPase inhibitor ouabain, and Gd^3+^ also had a partial effect, while other blockers showed no protective effect against cationic carriers, including the ROS scavenger butylated hydroxyanisole (Figure 4D and Supplementary information, Figure S4). Eosin is also the inhibitor of Na^+^/K^+^-ATPase, which binds to the ATP-binding site instead of cation-binding site on Na^+^/K^+^-ATPase, thus, eosin had little protective effect compared with ouabain. The concentration of ouabain is key to the inhibition of cell necrosis caused by cationic nanocarriers. Ouabain, at a rather low concentration, is enough to occupy the ouabain-binding site (OBS) of Na^+^/K^+^-ATPase without causing cell death; however, ouabain itself could induce necrotic cell death while given at a higher dose. These results made Na^+^/K^+^-ATPase (especially its OBS) a potential target in the interaction of cells with cationic nanocarriers that might also be responsible for the intracellular Na^+^ overload. However, no protective effect of ouabain was observed in the TNF-α induced necroptosis (Supplementary information, Figure S5).

To further address the role of Na^+^/K^+^-ATPase in cationic carrier-induced cell necrosis, cells were treated *in vitro* with cationic carriers for 5 min and heavy membrane fractions were prepared for determination of Na^+^/K^+^-ATPase activity. Na^+^/K^+^-ATPase activity was also assayed in crude homogenates from cationic carrier-treated mouse lungs. Both assays showed that there was a significant reduction in Na^+^/K^+^-ATPase activity in cells or tissues treated with cationic carriers while neutral and anionic carriers showed normal Na^+^/K^+^-ATPase activity level (Figure 4E and 4F). Furthermore, ^86^Rb^+^ uptake assay was carried out and inhibition of ^86^Rb^+^ uptake was observed in cells treated with cationic nanocarriers (Figure 4G). In addition, the pretreatment of mice with small dose of ouabain improved the survival after injection of fatal dose of cationic carriers (Figure 4H). These results indicated that treatment of cells with cationic carriers might inhibit cellular Na^+^/K^+^-ATPase activity, thus causing intracellular Na^+^ overload and subsequent cell necrosis.

To understand how cationic carriers mediated the impairment of Na^+^/K^+^-ATPase activity and to explain the protective effect of ouabain, we performed computational studies to simulate the interaction between cationic nanocarriers and Na^+^/K^+^-ATPase. Take DOTAP for instance, the calculation results indicate that, in the absence of ouabain, the hydrophobic tail of DOTAP might insert into an extracellular pocket of Na^+^/K^+^-ATPase that is the OBS. The positively-charged head of DOTAP (quaternary ammonium cation) locates at the entry of OBS pocket (Figure 4I). The calculated binding energy of DOTAP with Na^+^/K^+^-ATPase is −463.395 Kcal/mol, which is comparably as strong as the binding energy of pure ouabain with Na^+^/K^+^-ATPase (−504.262 Kcal/mol). We then calculated the binding energy between DOTAP and Na^+^/K^+^-ATPase with ouabain in OBS. The results showed that the binding energy of DOTAP with Na^+^/K^+^-ATPase decreased to −411.194 Kcal/mol. The similar calculations were also performed to simulate the interaction between Na^+^/K^+^-ATPase and PEI or chitosan. The calculated binding energies of the cationic nanocarriers with Na^+^/K^+^-ATPase in the absence or presence of ouabain are listed in Table 1. All of the results suggested that cationic nanocarriers could directly interact with the OBS of Na^+^/K^+^-ATPase. Interestingly, we also found that, at the same molarity, the cytotoxicity of cationic nanocarriers were correlated with the intensity of the binding energies with Na^+^/K^+^-ATPase (Supplementary information, Figure S6). Addition of ouabain might displace the cationic carrier from its preferred binding site, hence decreasing its binding energy with Na^+^/K^+^-ATPase, which provides a possible explanation for the protective effect of ouabain observed in the present study. Furthermore, we generated Na^+^/K^+^-ATPase knockdown A549 cell line and a reduction in the percentages of necrotic cells induced by cationic nanocarriers was observed when compared to control group (Figure 4J). In addition, transient receptor potential melastatin related 7 (TRPM7) was identified as one of the downstream targets of MLKL^32^, however, TRPM7 knockdown showed no protective effect against cell necrosis compared with control (Figure 4J and Supplementary information, Figure S7).

### Cationic carriers trigger pulmonary inflammation in mice through the induction of cell necrosis and release of mitochondria

**Figure 5:**
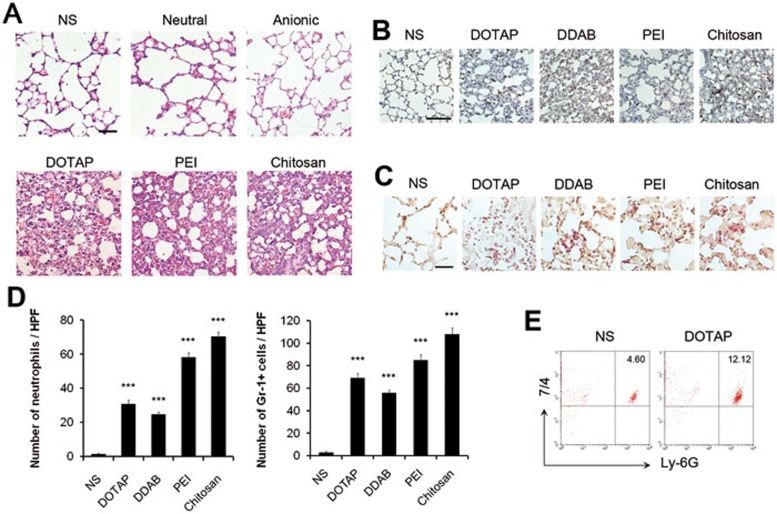
Pulmonary inflammation induced by cationic carriers upon systemic injection in mice. C57BL/6 mice were injected with various carriers: anionic, neutral liposomes (25 mg/kg), cationic DOTAP liposomes (25 mg/kg), PEI (5 mg/kg), Chitosan (25 mg/kg). *n* = 3 per group. Hematoxylin-eosin (HE) staining **(A)**, immunohistochemistry staining of Gr-1^+^ cells **(B)** and specific esterase staining of neutrophils **(C)** in representative mouse lung sections 24 h after injection are presented. Scale bar, 50 μm **(A**, **C)** and 200 μm **(B)**. **(D)** The stained Gr-1^+^ cells and neutrophils were counted in ten high power fields (HPFs). **(E)** Influx of neutrophils in whole lungs was detected after injection with flow cytometry by staining of 7/4 and Ly-6G. Data are mean ± SEM; *n* = 3.^***^P <0.001 compared with control group by Student's *t*-test.

Necrosis, also known as abnormal cell death, is considered to be responsible for the occurrence of subsequent sterile inflammation when induced *in vivo* in several diseases^29^. As the acute induction of cell necrosis by cationic nanocarriers was characterized, we postulated that the injection of cationic nanocarriers in mice might trigger subsequent inflammation. We found that mice injected with cationic nanocarriers through tail veins exhibited pulmonary inflammation, as shown by haematoxylin and eosin histology (Figure 5A). In contrast, mice injected with anionic and neutral liposomes showed normal pulmonary histology (Figure 5A). The inflammation appeared as early as 8 h after the injection which was hours after the induction of cell necrosis. Such pulmonary inflammation was characterized by infiltrates of neutrophil-like cells which were stained positive for Gr-1^+^ and esterase by histochemical staining and esterase staining, respectively. The positive cells in each high-power field (HPF) of mouse lung section were dramatically increased after injection of cationic liposomes, PEI and chitosan (Figure 5B-5D). Increase of neutrophils in lung tissue was also confirmed by flow cytometry (Figure 5E) with labeling of 7/4 and Ly-6G. In addition, inflammation characterized by infiltrates of neutrophil-like cells could also be found in the liver and other tissues to a lesser degree after injection of cationic nanocarriers (data not shown).

**Figure 6:**
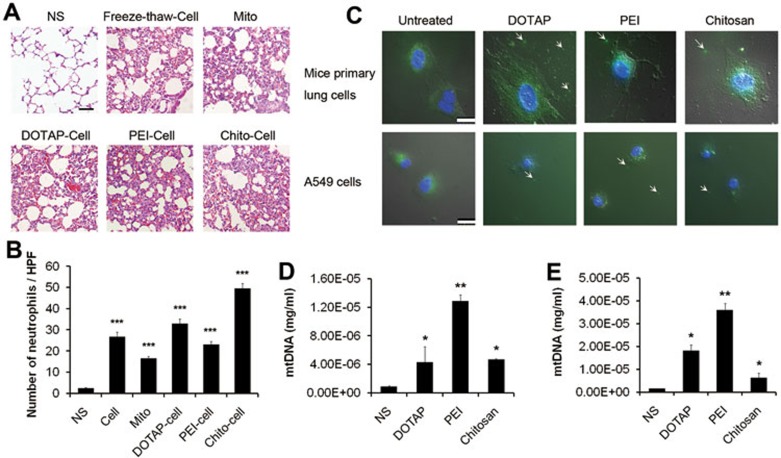
Pulmonary inflammation triggered by necrotic cells and the release of mitochondria. **(A**, **B)** Primary lung cells were either treated with DOTAP liposomes (50 μg/ml), PEI (10 μg/ml) and Chitosan (50 μg/ml) for 2 h to induce necrosis or treated with freeze-thaw process. Mitochondria were extracted from lungs of C57BL/6 mice. Treated cells and extracted mitochondria were injected into C57BL/6 mice (cells: 10^6^, mitochondria: 200 μg/mouse) for 24 h of exposure. HE staining of representative mouse lung sections was performed and the numbers of esterase-positive neutrophils in ten HPFs were counted. Scale bar, 50 μm. **(C)** Cells stained with Mito-tracker Green (mitochondria; green) and Hoechst 33342 (nucleus; blue) were treated with cationic carriers for 30 min at the concentrations above and observed for leakage of mitochondria. Scale bar, 25 μm. **(D)** Mice were injected with DOTAP liposomes (25 mg/kg), PEI (5 mg/kg) or Chitosan (25 mg/kg) and the mtDNA in serum was determined after 4 h of exposure by qPCR. *n* = 3 per group. **(E)** Primary lung cells were treated with cationic carriers at the concentration above for 4 h and supernatant was collected and concentrated for mtDNA determination. Data are mean ± SEM; *n* = 3.^*^P< 0.05,^**^P< 0.01,^***^P <0.001 compared with control group by Student's *t*-test.

Next, we investigated whether the inflammation was induced by necrotic cells triggered by cationic nanocarriers. Necrotic cells were prepared by incubating primary mouse lung cells with different cationic carriers, then washed and injected through mouse tail veins. Severe pulmonary inflammation was detected 24 h after injection (Figure 6A and 6B). Inflammation in lung was also observed in mice treated with necrotic cells induced by freeze-thaw process (Figure 6A and 6B). One reason for the induction of inflammatory response by necrotic cells is the release of DAMPs from necrotic cells which act as danger signals to alert the innate immune system^35^. DAMPs are known to consist of most intracellular components though, recently, mitochondria have emerged as one of the intracellular organelles that function as a source of DAMPs^37^. Inflammation induced by injection of extracted mitochondria in mice was also confirmed in our study (Figure 6A and 6B). Thus, we further considered that the leakage of mitochondria might play a role in the occurrence of pulmonary inflammation caused by cationic nanocarrier injection.

We detected the leakage of mitochondria from cationic nanocarrier-treated cells by staining cells with Mito-tracker. Mitochondria were released from necrotic cells after 30 min incubation with cationic nanocarriers (Figure 6C). In the meantime, the injection of cationic particles led to an increase in concentration of mtDNA in mouse serum as detected by real-time quantitative PCR (qPCR) (Figure 6D). Release of mtDNA was also confirmed in cells treated with cationic particles *in vitro* (Figure 6E). These findings suggest that mitochondrion and its DNA may be released from necrotic cells induced by cationic nanocarriers and mitochondrion itself could induce pulmonary inflammation after injection in mice.

### Neutrophil activation triggered by mtDNA and formyl-peptides

**Figure 7:**
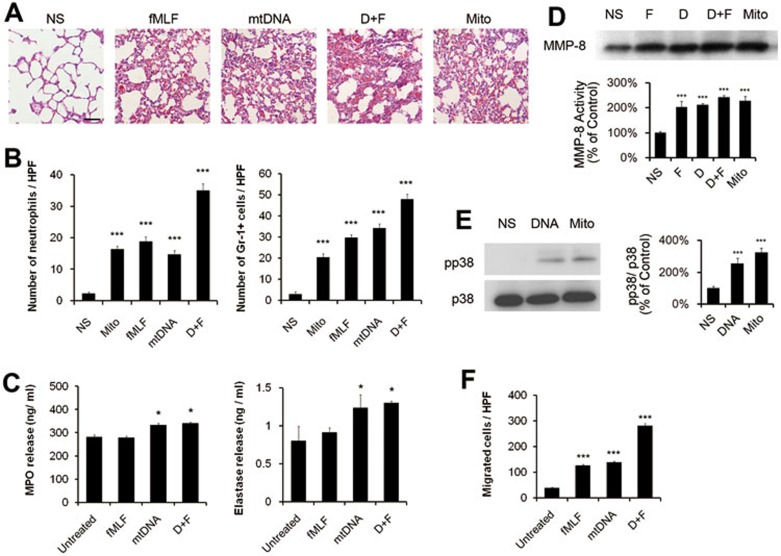
Neutrophil activation triggered by mtDNA and fMLF. **(A**, **B)** C57BL/6 mice were injected with normal saline, fMLF (1 μM, 200 μl), mtDNA (5 μg), mitochondria (200 μg) and sacrificed 24 h after injection. *n* = 3 per group. HE staining of representative lung sections was performed. Scale bar, 50 μm **(A)**. The esterase staining of neutrophils and the staining of Gr-1^+^ cells were counted in ten HPFs **(B)**. **(C**-**F)** Isolated murine neutrophils (5 × 10^6^) were treated *in vitro* for 2 h with fMLF (1 μM), mtDNA (5 μg/ml) and mitochondria (200 μg/ml). The release of MPO and elastase in the supernatant of the treated neutrophils was determined **(C)**. MMP-8 was determined by immunoblot in supernatants of the treated neutrophils (F: fMLF, D: mtDNA, D+F: mtDNA and fMLF, Mito: mitochondria). The relative intensity of MMP-8 was quantified **(D)**. Activation of p38 MAPK in neutrophils induced by mtDNA. Bar graph shows the relative intensity of pp38 to p38 **(E)**. Neutrophil chemotaxis assays were performed using a modified Boyden chamber with fMLF (1 μM) and mtDNA (5 μg/ml). The numbers of cells that migrated were counted in ten HPFs **(F)**. Data are mean ± SEM; *n* = 3.^*^P < 0.05,^***^P< 0.001 by Student's *t*-test.

We further studied the possible pathways by which released mitochondria trigger pulmonary inflammatory response. The injection of isolated mitochondria, mtDNA and synthetic peptide N-formyl-Met-Leu-Phe (fMLF) also caused severe inflammatory response in mouse lungs (Figure 7A and 7B). We measured the release of myeloperoxidase (MPO) and elastase from isolated mouse neutrophils in the culture supernatant after the treatment of mtDNA or fMLF. The addition of mtDNA induced more release of MPO and elastase from neutrophils, whereas fMLF showed minor effects compared with the control group (Figure 7C). In addition, mtDNA and fMLF also caused an increase in the release of matrix metalloproteinase-8 (MMP-8) from neutrophils which were shown by immunoblot with the supernatants (Figure 7D). The incubation of neutrophils with mtDNA or mitochondria activated p38 mitogen-activated protein kinase (MAPK) pathway. The addition of mtDNA or mitochondria to neutrophils caused phosphorylation of neutrophil p38 (Figure 7E). Furthermore, we assessed the effects of mtDNA and fMLF on neutrophil migration. As shown in Figure 7F, both mtDNA and fMLF stimulated neutrophil chemotaxis. Thus, mtDNA could activate neutrophil signaling and elicit an inflammatory neutrphil phenotype, which is proved by activation of p38 and increase in MPO release, respectively, and fMLF peptide may also be partially involved in some of the process.

### Induction of inflammation by cationic carriers through TLR9 pathway

**Figure 8:**
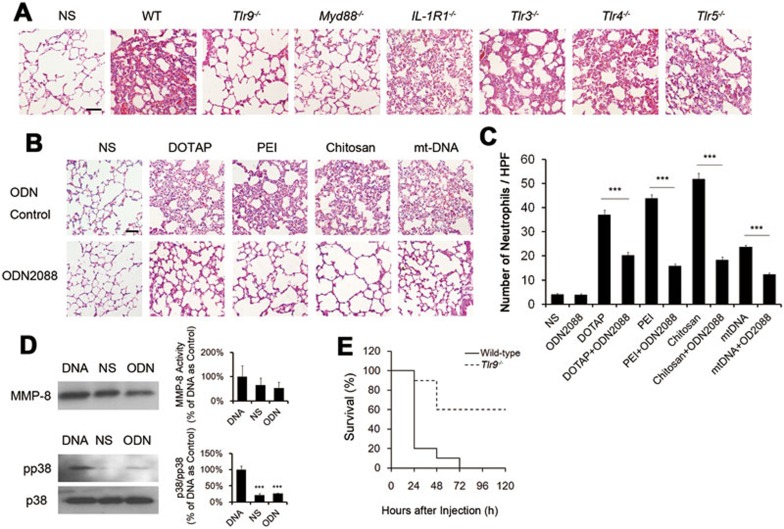
Important role of TLR9 pathway in necrotic cell-induced pulmonary inflammation. **(A)** Wild-type C57BL/6 mice and *Tlr9^−/−^*, *MyD88^−/−^*, *IL-1R1^−/−^*, *Tlr3^−/−^*, *Tlr4^−/−^*, *Tlr5^−/−^* mice were treated with mtDNA (5 μg/mouse) for 24 h and HE staining of lung sections was performed. Scale bar, 50 μm. **(B)** DOTAP liposomes (25 mg/kg), PEI (5 mg/kg), Chitosan (25 mg/kg) or mtDNA (5 μg/mouse) with ODN2088 or with ODN Control (50 μg/mouse) were injected into normal C57BL/6 mice. HE staining of mouse lung sections 24 h after injection is shown. Scale bar, 50 μm. **(C)** Positive cells in esterase staining of neutrophils were counted in ten HPFs. **(D)** Neutophils were treated with mtDNA (DNA, 5 μg/ml) or mtDNA and ODN2088 (ODN, 25 μg/ml) for 2 h. MMP-8 release and activation of p38 MAPK was examined by immunoblot in supernatants and cell pellets, respectively. The relative intensities of proteins were determined and shown in bar graphs. **(E)** Wild-type C57BL/6 mice and *Tlr9^−/−^* mice were injected with DOTAP liposomes (100 mg/kg) through tail veins every 24 h for 2 days and mouse survival were recorded every 24 h, *n* = 10. Data are mean ± SEM; *n* = 3.^***^P< 0.001 by Student's *t*-test.

As mtDNA might play an important role in the occurrence of inflammation induced by cationic carriers, we attempted to find the pathways through which mtDNA functions. We injected mtDNA into mice deficient in IL-1R1, various toll-like receptors (TLRs: TLR-3, TLR-4, TLR-5 and TLR-9) as well as MyD88. After the injection of mtDNA, *Tlr9^−/−^* and *MyD88^−/−^* mice showed reduction in pulmonary inflammation compared with wild-type mice, while others (*Tlr3^−/−^*, *Tlr4^−/−^*, *Tlr5^−/−^* and *IL-1R1^−/−^* mice) still had severe inflammatory response (Figure 8A). This implies that TLR9-MyD88 pathway may play an essential role in induction of inflammation by cationic liposomes or mtDNA. The injection of mitochondria in *Tlr9^−/−^* mice also showed reduction in pulmonary inflammation compared to wild-type mice (Supplementary information, Figure S8). Furthermore, we confirmed this finding by using the active TLR-9 antagonist ODN2088. Wild-type mice injected with ODN2088 (50 μg) together with cationic particles or mtDNA showed an reduction in neutrophil influx in lungs compared with normal saline and control ODN group (Figure 8B and 8C). Moreover, the pretreatment neutrophils with the inhibitory ODN2088 inhibited both the release of MMP-8 and activation of p38 MAPK induced by mtDNA (Figure 8D). Furthermore, the knockout of TLR9 in mice helped to improve the survival of mice injected with fatal dose of cationic carriers compared with the wild-type mice (Figure 8E).

## Discussion

Several observations have been made in this study concerning cationic nanocarriers, cell necrosis, pulmonary inflammation, mitochondrial DAMPs and the signaling mechanisms. Our results reveal a novel mechanism whereby cationic nanocarriers (including cationic liposomes, PEI, chitosan) induce acute cell necrosis through the interaction with Na^+^/K^+^-ATPase, with the subsequent exposure of mtDNA as a key event that mediates the inflammatory responses. This mechanism is supported by our findings. Namely, cationic particle-treated cells exhibited the morphological changes of the necrotic cells with swelled cytoplasm and ruptured plasma membrane. The PI-positive necrotic cells were also found in mouse lung tissues and BAL fluid of the mice after the injection of cationic carriers, before the occurrence of inflammation. The diffused cytoplasmic cathepsin-B immunofluorescence was observed without the activation of caspase in apoptosis. The cell membrane depolarization was detected in less than 60 s of treatment, which occurred before the earliest cell necrosis. A linear regression analysis showed a positive correlation between the zeta-potential of cationic liposomes and the percentages of induced necrotic cell death. The cell necrosis was associated with intracellular Na^+^ overload and depletion of Na^+^ in culture medium significantly decreased the percentages of necrotic cells induced by cationic nanocarriers. The impairment of Na^+^/K^+^-ATPase was then characterized as one of the reasons for Na^+^ overload and cell necrosis. Cell necrosis could be apparently decreased by pretreatment of cells with Na^+^/K^+^-ATPase inhibitor ouabain, which functions by binding the cation-binding sites on Na^+^/K^+^-ATPase. The computational simulation showed that cationic carriers could directly interact with OBS of Na^+^/K^+^-ATPase (cation-binding site) and the addition of ouabain displaced the cationic carrier from its preferred binding site. Also, a small dose of ouabain could effectively extend the survival of mice which received the fatal dose of cationic nanocarriers. The Na^+^/K^+^-ATPase knockdown cell line showed a reduction in the percentage of cell necrosis induced by cationic nanocarriers compared with control cell line and TRPM7-knockdown cell line. The pulmonary inflammation was caused by systemic injection of cationic carriers in mice. The release of mitochondria was observed in cationic carrier-treated cells by Mito-tracker staining and the concentration of mtDNA was increased in the serum of mice injected with cationic carrier as detected by qRT-PCR. The injection of the necrotic cells, the isolated mitochondria as well as mtDNA and peptide N-formyl-Met-Leu-Phe (fMLF), could also induce severe inflammation in mouse lungs. Neutrophil activation was characterized by increased release of MPO and elastase triggered by mtDNA. In addition, the release of MMP-8 and activation of p38 MAPK pathway were also observed. The activation of neutrophils by mtDNA through TLR9 pathway was confirmed by using *TLR9^−/−^* mice and TLR-9 antagonist ODN2088, and the inflammation was reduced in both *TLR9^−/−^* and ODN2088-treated mice. On the basis of our findings mentioned above, we may also rule out the possibility that the cell necrosis detected in the lung tissues and BAL fluid may result from the inflammatory microenvironment.

It has been reported that typical cationic carriers such as cationic liposome, chitosan and PEI can trigger cell apoptosis^41,42^. The induction of these apoptotic cells often occurred after approximately 24-48 h of the treatment^41,42^. These findings were confirmed in our study. However, we found that these cationic carriers triggered an additional cell death, acute cell necrosis, before the occurrence of cell apoptosis. These necrotic cells were characterized by the rapid cell membrane damage which was detected by PI staining as early as 5 min in the cells treated with cationic carriers. The cells exhibited necrotic morphology with swollen granular cytoplasm and ruptured plasma membrane. The diffuse cytoplasmic release of cathepsin-B was also observed which is generally considered a marker of necrosis for the loss of lysosomal membrane integrity. No features of apoptotic cell death were found in the cells treated with cationic nanocarriers in minutes, whereas after 24 h of incubation with cationic nanocarriers, the cells were characterized by the apoptotic changes with increase in sub-G1 proportions and nuclear fragmentation. Necrosis, also known as abnormal cell death, is postulated to release the immunostimulatory DAMPs to trigger sterile inflammation, whereas apoptotic cells generally do not induce an inflammatory response^29^. In addition, we found in the present study that the necrotic cells, their mtDNA release and the following inflammation all appeared before the occurrence of cell apoptosis. Thus, these findings indicated that the inflammation observed in the present study before apoptosis, may mainly result from the necrotic cells.

Cell necrosis was generally characterized as abnormal cell death induced by unavoidable damages, which could also be tightly controlled by intrinsic cellular programs and named as “necroptosis”, such as the cell necrosis involving RIP1 and Mlkl pathways^30,31,32,33,43^. However, in this study, cationic carrier-induced cell necrosis does not require either RIP1 or Mlkl, but is related to the surface cationic charge of the nanocarriers. One reason for this is that cationic carriers could specifically impair the activity of Na^+^/K^+^-ATPase on cell membrane, which contributes to the subsequent intracellular Na^+^ overload and finally results in cell necrosis. In support of this conclusion, evidences are as follows. The intracellular Na^+^ overload was monitored by staining cells with CoroNa Green and the depletion of Na^+^ in culture medium significantly decreased the number of necrotic cells induced by cationic carriers. The activity of Na^+^/K^+^-ATPase in homogenates were inhibited after treating cells or mice with cationic nanocarriers. It is interesting to find that after the pretreatment of cells with Na^+^/K^+^-ATPase cation-binding site inhibitor ouabain, the cells are apparently protected from necrosis induced by cationic carriers, whereas the Na^+^/K^+^-ATPase ATP-binding site inhibitor eosin showed no protective effect. The Na^+^/K^+^-ATPase knockdown cell line also confirmed the protective effect with a reduction in the percentage of cell necrosis induced by cationic nanocarriers compared with wild-type cell line and TRPM7-knockdown cell line. Mice pretreated with small dose of ouabain had improved survival after the injection of fatal dose of cationic nanocarriers compared with control group. Also, the computational simulation showed that cationic carriers could directly interact with OBS of Na^+^/K^+^-ATPase with a rather strong binding energy and the addition of ouabain displaced the cationic carrier from its preferred binding site. The resulting inhibition of Na^+^/K^+^-ATPase activity caused by cationic nanocarriers also exhibited a similar cellular biological effect to that induced by high dose of ouabain, such as depolarization of cell membranes and subsequent cell necrosis. However, when small dose of ouabain first occupies OBS, it significantly weakens the interaction between cationic carriers and Na^+^/K^+^-ATPase and results in a protective effect of ouabain. All these results support our conclusion that the impairment of Na^+^/K^+^-ATPase activity and the intracellular Na^+^ overload were responsible for cell necrosis induced by cationic nanocarriers. These findings brought new evidence for how the cationic potential of a particle affects the cell biological behavior. Therefore, care needs to be taken during the evaluation of early toxicity of the cationic particles.

It was reported that ouabain interact with the Na^+^/K^+^-ATPase cation-binding site, disrupting the Na^+^/K^+^-ATPase activity^44^. The investigation on the interaction between ouabain and Na^+^/K^+^-ATPase revealed that the substitutions of Thr-797 residue in Na^+^/K^+^-ATPase protein resulted in 66-79-fold reduction in the inhibitory effect of ouabain on Na^+^/K^+^-ATPase activity. The researchers assumed that the binding of ouabain to such a region blocks the movement of the H5 and H6 transmembrane domains which may be required for both energy transduction and cation transport^44^. Thus, these findings could help to explain why cationic nanocarriers which interact with the cationic-binding site cause the sodium overload and also disrupt the Na^+^/K^+^-ATPase activity.

Cell injury could release the intracellular DAMPs which are recognized by recognition receptors. Released DAMPs alert the innate immune system and result in a sterile inflammatory response, such as trauma and atherosclerosis^37,45^. Recently, mitochondria have emerged as one of the intracellular organelles that function as a source of DAMPs^38,39^. Due to the hypothesis that mitochondria probably originated from α-Protobacteria more than a billion years ago, mitochondria are often considered as “dangerous organelles” which still possess many features of their bacterial ancestors. The circular genome containing CpG DNA and the ability to form N-formyl peptides are mainly responsible for its immunostimulatory effect^37,38,39^. Thus, mitochondrial DAMPs can modulate immune responses and contribute to the development of inflammatory diseases. These findings may help to explain the findings in the present study that the observed inflammation was induced by the release of mtDNA and fMLF from cationic carrier-treated necrotic cells.

Since it has been known that besides mitochondrial contents, the other DAMPs such as HMGB1 (high mobility group box 1), IL-1a, uric acid, may also be released from necrotic cells^39^, whether these non-mitochondrial DAMPs may also play a role in the inflammatory response mediated by cationic nanocarriers is an intriguing question to be further explored. However, the present findings indicate that the release of mtDNA and fMLF from the mitochondria of the cationic carrier-treated necrotic cells may play an important role in the induction of inflammation. This conclusion is drawn from the following experimental data in the present study: (1) Necrotic cells induced by cationic carrier released the mitochondria and its DNA; (2) mtDNA and formyl-peptides triggered neutrophil activation; (3) the pretreatment of neutrophils with the active TLR-9 antagonist ODN2088 inhibited both the release of MMP-8 and activation of p38 MAPK induced by mtDNA; (4) *Tlr9^−/−^* and *MyD88^−/−^* mice showed reduction in the inflammation; (5) TLR-9 antagonist ODN2088 inhibited the inflammation and *Tlr9^−/−^* mice showed extended survival after injection of cationic carriers.

Taken together, in the present study we find that the cationic nanocarriers could induce acute cell necrosis. We further provide a perspective on the new mechanism for the cell necrosis and a new explanation for the subsequent occurrence of inflammatory response caused by cationic carriers. The understanding of acute cell damage caused by cationic carriers may assist in the design of nanocarriers in DDSs and provide insight into the development of screening assays and rapid assessment of biomaterials. This might be of great importance to evaluate the biocompatibility of the nanocarriers and design better and safer ones for drug delivery.

## Materials and Methods

### Cells and animals

Cells were obtained from American Type Culture Collection. Mouse primary lung cells were isolated and cultured with a modified method as described previously^46^. *Tlr3^−/−^*, *Tlr4^−/−^*, *Tlr5^−/−^*, *IL-1R1^−/−^* and *MyD88^−/−^* mice with C57BL/6 background were obtained from The Jackson Laboratory. *Tlr9^−/−^* mice with C57BL/6 background were obtained from Bioindustry Division Oriental Yeast Co., Ltd. (Tokyo, Japan). All animal experiments are approved by the Institutional Animal Care and Use Committee and Ethics Committee.

### Liposome preparation

Cationic, anionic and neutral liposomes were all prepared by conventional evaporation method^47^. Cationic liposomes were made of either N-[1-(2,3-Dioleoyloxy)propyl]-N,N,N-trimethylammonium chloride (DOTAP-Cl)/dioleoyl phosphatidylethanolamine (DOPE) or dimethyldidodecylammonium bromide (DDAB)/DOPE or DOTAP-Cl alone. Anionic liposomes were formed by the addition of phosphatidylcholine (PC), cholesterol (Chol) and anionic cholesteryl hemisuccinate. Neutral liposomes consist of PC and Chol. PEI (25k, Sigma) was used as received. N-trimethyl chitosan free carriers were modified from Chitosan (deacetylation of 75%-85%, Medium molecular weight, Sigma)^48^. The size distribution and zeta-potential of the prepared liposomes were determined by Malvern Nano-ZS 90 laser particle size analyzer. The concentrations of the liposomes were 1 mg/ml and dispersed in water at room temperature during the measurement of size distribution and zeta-potentials. The particle sizes of prepared liposomes were in the range of 100-150 nm with zeta-potentials varied with the components. Liposomes with different zeta-potentials were prepared by adjusting the ratio of DOTAP/DOPE. In the charge-neutralization experiment, liposomes were mixed with different concentrations of BSA for 5 min to block the surface charge before adding to the cells.

### Flow cytometry

For the detection of necrotic cell, cells were labeled with Annexin-V-FLUOS (Roche Diagnostics) according to the manufacturer's instruction. For the BAL fluid, cells were harvested, counted and dispersed at the same concentration in dHBSS^49^. Analysis was carried out on a FACS Calibur flow cytometer (BD Biosciences). For the detection of neutrophil infiltrates in lungs, lung tissues were excised and cut into small pieces (∼5 mm) and incubated with Collagenase I at 37 °C for 2 h. Cells were washed twice with PBS, counted and dispersed in PBS at 10^6^ cell/ml and stained with FITC-rat anti-mouse 7/4 (AbD Serotec, 1:100) and PE-rat anti-mouse Ly6G (BD Biosciences, 1:100). The oxonol dye diBA-C4-(3) (Sigma) at a concentration of 100 nM was used for the membrane potential (ψ) measurements by flow cytometry as previously reported^50^.

For the measurement of sub-G1 fraction by PI, cells were treated with DOTAP for the indicated time and the harvested cells were washed with PBS, fixed with 70% ethanol at −20 °C for 30 min and washed. Cells were transferred into staining solution (20 μg/ml PI, 5 μg/ml ribonuclease A, 0.1% Triton X-100 in PBS) and incubated at room temperature for 10 min before detection.

For the cell viability assay in sodium-free medium, cells were exposed to experimental conditions in Krebs-Ringer-HEPES buffer (136 mmol/l NaCl, 20 mmol/L HEPES, 4.7 mmol/l KCl, 1.25 mmol/l MgSO_4_, 1.25 mmol/l CaCl_2_, pH 7.4) supplemented with 25 mmol/l glucose (KRH-glc) with Na^+^ placed equimolarly by N-methyl-D-glucamine as reported^51^.

### Immunohistochemistry and esterase staining

Three-micrometer sections of the selected formalin-fixed, paraffin-embedded specimens were prepared and Gr-1 staining was performed using an anti-mouse Gr-1 Ab (BD Biosciences, 1:50). Esterase was stained with Naphthol AS-D Chloroacetate Kit (Sigma) and procedure was performed according to the manufacturer's instruction. Stained slides were examined using an upright microscope (Eclipse 80i, Nikon) and the positive cells were counted in high-power fields (HPFs; 400×).

### Fluorescence microscopy

Immunofluorescent study of A549 cells was performed with cathepsin B antibody (Abcam, 1:100) and visualized by an upright microscope (Eclipse 80i, Nikon).

For the monitoring of intracellular sodium level, cells were first loaded with CoroNa Green (Invitrogen) and PI according to the manufacture's protocol. Then, cationic carriers were added and fluorescence was monitored by a fluorescent microscope. Images were recorded every 60 s. The fluorescent intensity was analyzed using an image analysis program (Image J).

To detect the release of mitochondria caused by cationic carriers, the mouse primary lung cells and A549 cells were stained with Mito-Tracker Green (Beyotime Institute of Biotechnology, Nantong, China) at a final concentration of 100 nM and Hoechst 33342 (20 μg/ml, Sigma) for 30 min. Then, cells were treated with various cationic carriers at the indicated concentration for another 2 h. The supernatant was removed gently and cells were observed under the Leica TCS SP5 confocal microscope.

### Detection of necrosis *in vivo* by PI

Nanocarriers were injected into the tail vein of mice. 0.5 μl of PI (Sigma; 1 mg/ml in normal saline) was injected into tail vein 2 h later. 5 min after injection of PI, mice were perfused with 4% formaldehyde both through tail vein and into thoracic cavity, as described^52^. Then, lung was removed and several sections from each animal were examined with necrotic cell death markers PI on frozen (4-μm-thick) sections.

### BAL

BAL was performed as described^49^.

### Determination of intracellular Ca^2+^ and ROS

Intracellular Ca^2+^ and ROS were measured with H_2_DCF-DA and Fluo-3/AM, respectively^52^.

### MTT assay

MDFs were isolated from three *Mlkl^−/−^* mice and three congenic wild-type mice. MTT assays were carried out in 96-well plates (seeding 10^4^ MDFs cells). Cells attached overnight and then were treated with different combinations of QVD-OPH (5 mM), TNF (100 ng/ml) and Smac mimetic (500 nM) as well as ouabain. After 24 h of treatment, the fluid was replaced with new culture medium containing MTT (Sigma, 0.5 mg/ml) and the cells were incubated for 4 h. Then, 150 μl DMSO was added to dissolve the precipitate. Absorbance was measured by spectrophotometer at 570 nm using a microplate reader. Each concentration was replicated in 6 wells. The blank group and control group were set up simultaneously.

### Determination of Na^+^/K^+^-ATPase activity

Na^+^/K^+^-ATPase activities of cationic carrier-treated tissue and cells were measured by coupling ADP production to oxidation of NADH and recording the absorbance change at 340 nm as previously described^51^. Mice were injected with cationic nanocarriers through tail veins and sacrificed. Then, 20 mg lung from each animal was collected, washed and homogenized on ice in reaction medium. For the *in vitro* evaluation, A549 cells were treated with cationic carriers before the induction of cell necrosis, and harvested, washed and homogenized on ice in reaction medium. The heavy membrane fractions were collected for assays and prepared as described^53^. After the homogenization, intact cells and nuclei were removed by centrifugation at 500× *g* for 10 min at 4 °C. The supernatant was subjected to centrifugation at 10 000× *g* for 15 min at 4 °C to obtain the heavy membrane fraction (pellet). Protein content of heavy membrane fraction was determined and Na^+^/K^+^-ATPase activity was assayed using the same protein amount of heavy membrane fraction. The reaction medium contained 1 mM phosphoenolpyruvate, 5 mM MgC1_2_, 120 mM NaCl, 25 mM KCl, 0.1 mM EGTA, 50 mM triethanolamine, pH 7.4. 0.3 mM NADH, 4 units/ml pyruvate kinase, 20 units/ml lactic dehydrogenase and 2.5 mM MgATP were added just before use. Reaction was carried out with or without pretreatment of sample with ouabain (1 mM) for 1 h. Na^+^/K^+^-ATPase activity was calculated by subtracting the amount of ouabain-insensitive ATPase activity from the total ATPase activity. All reagents were purchased from Sigma. NADH absorbance was measured using NanoDrop ND-1000 (NanoDrop Technologies). Reaction buffer was used as negative control.

### Rubidium uptake assay

A549 cells were cultured in 6-well plates overnight (5 × 10^5^ cells/well). Cells were treated with DOTAP liposome (50 μg/ml), PEI (10 μg/ml), chitosan (50 μg/ml), anionic (50 μg/ml) and neutral liposomes (50 μg/ml) for 2 min, respectively, followed by directly adding ^86^Rb^+^ (PerkinElmer; final concentration, 5 μCi/ml) to each culture and ^86^Rb^+^ uptake was terminated by sucking off the medium. Cells were washed three times with ice-cold PBS and extracted with 1 ml 10% trichloroacetic acid for 1 h. The extract was added to 10 ml water to measure Cerenkov radiation by the scintillation counter (Ludlum Measurements, Inc.). Radioactive counts were normalized by protein content in each corresponding sample.

### Computational details

All the computational studies were carried out using Discovery Studio (DS; Accelrys, San Diego, CA). The binding energy (ΔE) of DOTAP with Na^+^/K^+^-ATPase was calculated according to eq 1.


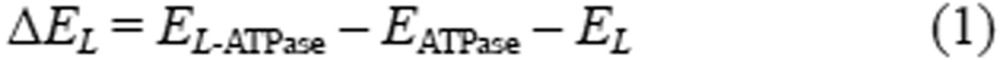


Where L is DOTAP. *E_L_* represents the total energy of X.

For the calculation of *E*_ATPase_, a conventional molecular dynamics (MD) simulation was performed in advance for the ATPase protein. The average structure of ATPase protein in the final 10 ps trajectory (thermodynamic equilibration state) was used to calculate the total energy. For the calculation of *E*_*L*−ATPase_, the ligand L was firstly docked into the binding site of ATPase protein, followed by a similar MD simulation as above. *E*_L_ was calculated based on the minimum energy conformation of L, which was optimized by a force-field method.

The initial structures for ATPase and OUABAIN-ATPase complexes were prepared based on the X-ray crystal structures (PDB: 4HYT). The unresolved residues were constructed by using the homology modeling module Modeler within DS.

In dynamic simulations, constant molecular number, pressure and temperature and periodic boundary conditions were used. The CHARMM force field was employed for the protein and the gaff force field for small molecules. By assuming normal charge states of ionizable groups corresponding to pH 7, sodium (Na^+^) and chloride (Cl^−^) counter-ions at physiological concentration of 0.15 mol/L were added in the box in random positions to ensure the global charge neutrality. All Na^+^ and Cl^−^ ions were placed more than 8 Å away from any protein atoms and from each other. Constant temperature (T = 310 K) and constant pressure (P = 1 bar) were maintained using Langevin piston coupling algorithm. The integration time step of the simulations was set to 2.0 fs, the SHAKE algorithm was used to constrain the lengths of all chemical bonds involving hydrogen atoms at their equilibrium values, and the water geometry was restrained rigidly by using the SETTLE algorithm. Non-bonded van der Waals interactions were treated by using a switching function at 10 Å and reaching zero at a distance of 12 Å. Long-range electrostatic forces were handled by using the particle-mesh Ewald algorithm, which is an efficient full electrostatics method for use with periodic boundary conditions. The systems were firstly minimized using steepest-descents algorithm. Afterward, the systems were heated from 0 K to 310 K in two steps: the first step was a 100 ps heating process with the protein and ligand fixed; the second step was another 100 ps heating process with the whole system relaxed. Thirdly, a 10 ps CMD simulation was performed with the protein and ligand fixed, followed by a 10 ps CMD simulation with the whole system relaxed.

### Generation and validation of Na^+^/K^+^-ATPase and TRPM7 knockdown cell line

The shRNA plasmid against human Na^+^/K^+^-ATPase, alpha 1 polypeptide (ATP1A1, GenBank accession number NM_000701) and the shRNA plasmid against human transient receptor potential cation channel, subfamily M, member 7 (TRPM7, GenBank accession number NM_017672) were purchased from Sigma-Aldrich. Packing vectors (pMD2.0G and psPAX) with either scramble or shRNA plasmids were transfected into HEK293T cells for 24 h and supernatant was collected. The lentiviral preparations were used to infect A549 cells. After 24 h of infection, cells were selected with puromycin for another 48 h. Knockdown of ATP1A1 and TRPM7 in the selected cells were confirmed by real-time PCR and western blotting. Total RNA was extracted using TRIzol Reagent (Cat# 15596026, Life Technologies, Frederick, MD, USA). Then 1 μg of total RNA was used to synthesize first-strand cDNA with a PrimeScript RT reagent kit and gDNA Eraser (RR047A, TaKaRa Bio Inc., Dalian, China) according to the manufacturer's instructions. Quantitative real-time PCR was performed using SsoAdvanced SYBR green supermix (172-5260, Bio-Rad) in a BioRad CFX-96 real time system. The specific primers employed are as follows: TRPM7, 5′-TAGCCTTTAGCCACTGGAC-3′, 5′-GCATCTTCTCCTAGATTTGC-3′ ATPA1, 5′-GGGAAGGGGGTTGGACG-3′, 5′-CCGGCTCAAGTCTGTTCCAT-3′ GAPDH, 5′-CACCATCTTCCAGGAGCGAG-3′, 5′-CTTCTCCATGGTGGTGAAGAC-3′. Relative expression of each gene was normalized to that of GAPDH protein. For western blot assay, antibodies to ATPA1 and TRPM7 were purchased from Abcam and were used as 1:200 and 1:500, respectively.

### Preparation of mitochondria and mtDNA

The Qproteome Mitochondria Isolation Kit (Qiagen) was used to isolate mitochondria from mouse lungs. Mitochondria were isolated under sterile conditions at 4 °C and kept at −80°C for long-term storage. Protein concentrations were determined using the Coomassie Brilliant Blue G-250 method. To mimic the *in vivo* release of mitochondria and mtDNA, freeze-thaw process was added before the use to disrupt the mitochondria membrane. The isolation of mtDNA was carried out using mtDNA isolation Kit (Abcam). mtDNA concentration was determined by a spectrophotometer. No protein contamination was found.

### Quantitative real-time PCR for mtDNA

The mtDNA in plasma and in cell culture supernatant was concentrated and purified using QIAamp DNA Blood Mini Kit (Qiagen).

The quantitation of mtDNA was performed with Taqman probes. The PCR primers and probes used to amplify the mouse mtDNA were designed, referring to the GenBank nucleotide sequence (J01420, Mus musculus mitochondrion genome, positions 2891-3173)^54^. Primers were designed and synthesized by Invitrogen. Sense primer, 5′-ACCTACCCTATCACTCACACTAGCA-3′, antisense primer, 5′-GAGGCTCATCCTGATCATAGAATG-3′, FAM-labeled TAMRA-quenched probes, 5′-ATGAGTTCCCCTACCAATACCACACCC-3′. The standard curve was created by analyzing serial dilutions of plasmid DNA inserted with the target PCR product (J01420, positions 2891-3173).

### Isolation of mouse bone marrow neutrophils, mouse peritoneal macrophages and lymphocytes

Mouse neutrophils were prepared from isolated bone marrow as previously described^55^. More than 85% of the pelleted cells were neutrophils as determined by flow cytometry.

Mouse peritoneal macrophages were isolated by injecting 5 ml of sterile PBS into mouse peritoneal cavity followed by a gentle shake of the entire body for 10 s, then, slow withdrawal of saline containing resident peritoneal cells by inserting a 19-gauge needle. Cells were washed and placed into incubator for 45 min to allow macrophages to adhere.

Mouse lymphocytes were isolated by cutting the spleen into small pieces and squeezing the splenocytes through the 70-μm nylon mesh of the cell strainer to create a single cell suspension. Cell suspension was carefully poured into the ficoll solution and centrifuged at 1 600 rpm for 20 min and the separated lymphocytes were collected.

### Myeloperoxidase production and elastase release

The isolated mouse bone marrow neutrophils were placed in a 24-well plate at a density of 5 × 10^6^ cells in 1 ml of dHBSS. fMLF (1 μM), mtDNA (5 μg/ml) were added to the cells and incubated for 4 h. MPO released into culture medium was determined using EnzChek Myeloperoxidase Activity Assay Kit (Invitrogen). Release of neutrophil elastase was measured by enzyme-linked immunosorbent assay (ELISA) using Mouse neutrophil elastase ELISA Kit (CUSABIO Life science, China).

### Chemotaxis assay

Chemotaxis assay was performed using multiwall chambers separated by a 3 μm polyhydrocarbon filters as described previously^56^. The concentrations of fMLF and mtDNA are 1 μM and 5 μg/ml, respectively. Cells that migrated across the filter were dehydrated, fixed, and stained with hematoxylin. The stained cells in ten randomly chosen HPFs (400×) in that well were then counted.

### Western blot assay

The freshly isolated neutrophils were resuspended in dHBSS at a concentration of 5 × 10^6^ cell/ml. The indicated reagents were added to the cells and incubated for 2 h at 37 °C. After the incubation, 1 ml supernatant was collected and concentrated by centrifugation through Amicon Ultra-0.5 (Millipore) to the same final volume of 50 μl. The residual neutrophil pellet was lysed and protein samples were prepared. Antibodies to phospho-p38 MAPK (Thr 180/Tyr 182; Cell Signaling), p38 MAPK (Cell Signaling) and MMP8 (Abcam, 1:1 000 for all) were used.

## Figures and Tables

**Table 1:** The binding energy of cationic carriers with Na^+^/K^+^-TPase without or with ouabain in OBS

Ligand	ΔE (Kcal/mol)	ΔE_OBN (Kcal/mol)
DOTAP	−463.195	−411.194
PEI	−1894.947	−1065.309
Chitosan	−302.027	−231.497

## Supplementary Material

Supplementary information, Figure S1 Pulmonary inflammation induced by cationic liposomes and lipoplexes upon systemic injection.

Supplementary information, Figure S2 Acute cell necrosis induced by cationic carriers in normal cells.

Supplementary information, Figure S3 Apoptotic cell death appeared after 24 hours of treatment of cells with cationic carriers.

Supplementary information, Figure S4 No reduction in cell necrosis after the pretreatment of butylated hydroxyanisole (BHA).

Supplementary information, Figure S5 No inhibitory effect of ouabain in TSQ-induced cell necroptosis.

Supplementary information, Figure S6 Cell necrosis induced by cationic nanocarriers of equal molarity.

Supplementary information, Figure S7 The knockdown of ATPA1 and TRPM7 in A549 cell line.

Supplementary information, Figure S8 The reduction of pulmonary inflammation induced by mitochondria in *Tlr9^−/−^* mice.
