# Supplementary material for: Cationic nanocarriers induce cell necrosis through impairment of Na+/K+-ATPase and cause subsequent inflammatory response
Source: Cell Res. 2015 Jan 23;25(2):237–53. doi: 10.1038/cr.2015.9 (PMC4650577; doi:10.1038/cr.2015.9)
Supplement: Supplementary information, Figure S1 — Pulmonary inflammation induced by cationic liposomes and lipoplexes upon systemic injection. [file cr20159x1.pdf]

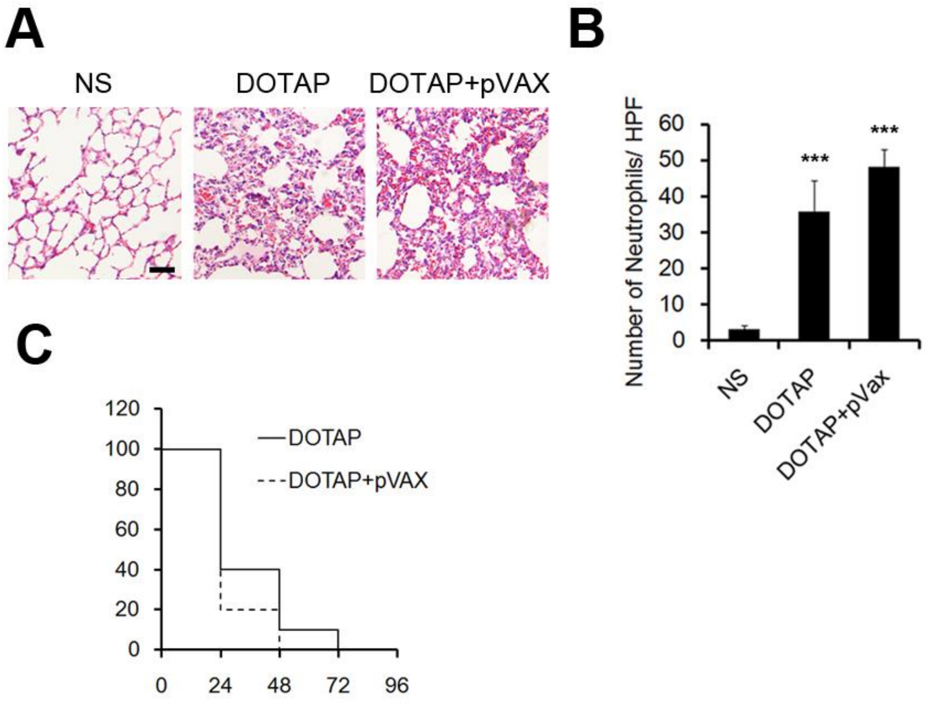

Supplementary information, Figure S1 Pulmonary inflammation induced by cationic liposomes and lipoplexes upon systemic injection.

(A) C57BL/6 mice were injected with DOTAP liposomes (25mg/kg) and DOTAP lipolexes with pVAX plasmid loaded (25mg/kg, DNA/liposome ratio, 1/10). *n*=3 per group. HE staining in representative mice lung sections 24 h after injection was performed. Scale bar, 50 μm. **(B)** Neutrophils in each section were stained by esterase staining and were counted in ten high power fields (HPFs). Data are mean ±SEM; *n*=3. $^{***}P<0.001$ compared with control group by Student's *t*-test. **(C)** Mice were injected with DOTAP liposomes (100mg/kg) or DOTAP lipsoplexes (100mg/kg, DNA/liposome ratio, 1/10) through tail veins every 24 h for two days and mice survival were recorded every 24 h, *n*=10.
