# Supplementary material for: Cationic nanocarriers induce cell necrosis through impairment of Na+/K+-ATPase and cause subsequent inflammatory response
Source: Cell Res. 2015 Jan 23;25(2):237–53. doi: 10.1038/cr.2015.9 (PMC4650577; doi:10.1038/cr.2015.9)
Supplement: Supplementary information, Figure S2 — Acute cell necrosis induced by cationic carriers in normal cells. [file cr20159x2.pdf]

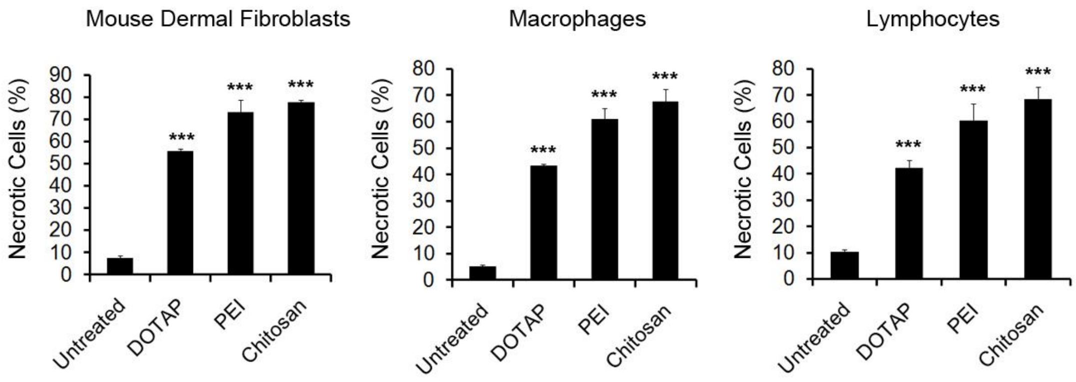

Supplementary information, Figure S2 Acute cell necrosis induced by cationic carriers in normal cells.

Normal primary cells including mouse dermal fibroblasts, peritoneal macrophages and splenic lymphocytes were prepared and treated with DOTAP liposome (50μg/ml), PEI (10μg/ml) and Chitosan (50μg/ml) for 10 min. The necrotic cells were detected by flow cytometry with Annexin-V and PI staining. Data are mean±SEM; n=3. $^{***}P$<0.001 by Student's t-test.
