# Supplementary material for: Cationic nanocarriers induce cell necrosis through impairment of Na+/K+-ATPase and cause subsequent inflammatory response
Source: Cell Res. 2015 Jan 23;25(2):237–53. doi: 10.1038/cr.2015.9 (PMC4650577; doi:10.1038/cr.2015.9)
Supplement: Supplementary information, Figure S3 — Apoptotic cell death appeared after 24 hours of treatment of cells with cationic carriers. [file cr20159x3.pdf]

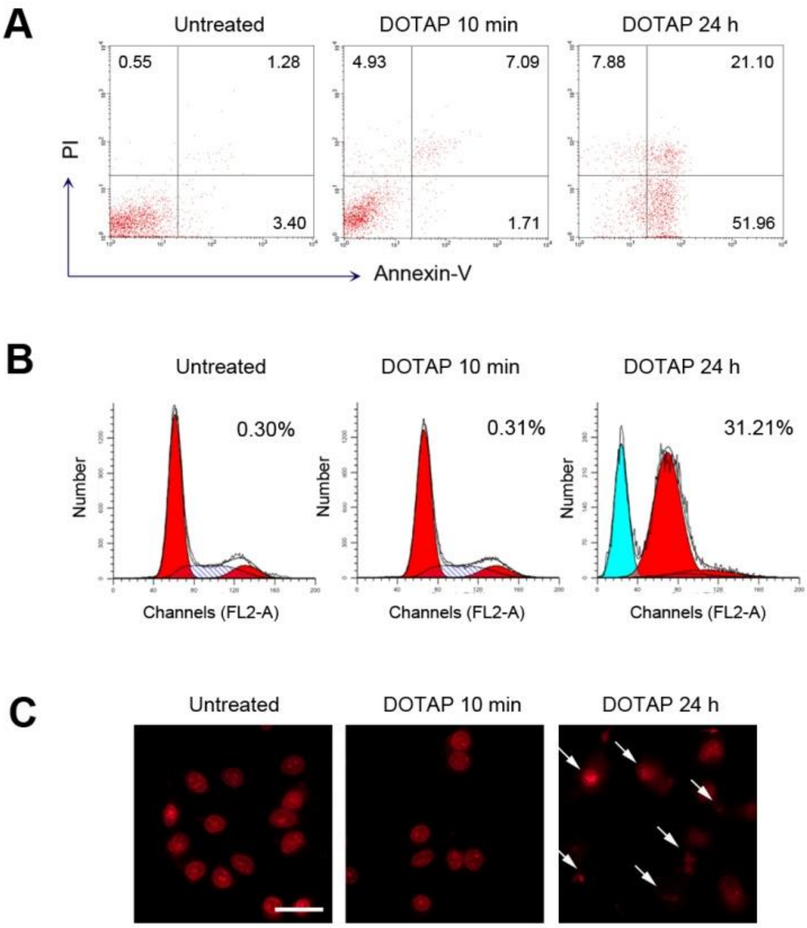

Supplementary information, Figure S3 Apoptotic cell death appeared after 24 hours of treatment of cells with cationic carriers.

(A) Annexin-V-positive apoptotic cells were increased after 24 hours of treatment of A549 cells with DOTAP liposomes (25μg/ml). Numbers in each quadrant indicate the percentage of gated cells. **(B)** Apoptotic cells were increased after 24 hours of treatment of A549 cells with DOTAP liposomes (25μg/ml). The sub-G1 fraction (apoptotic cells) was detected by flow cytometry. The percentages of sub-G1 fractions are given. **(C)** The nuclear fragmentation was observed in cells after 24 h of treatment of A549 cells with DOTAP liposomes (25μg/ml) by staining with PI; Scale bar, 20 μm.
