# Supplementary material for: Cationic nanocarriers induce cell necrosis through impairment of Na+/K+-ATPase and cause subsequent inflammatory response
Source: Cell Res. 2015 Jan 23;25(2):237–53. doi: 10.1038/cr.2015.9 (PMC4650577; doi:10.1038/cr.2015.9)
Supplement: Supplementary information, Figure S4 — No reduction in cell necrosis after the pretreatment of butylated hydroxyanisole (BHA). [file cr20159x4.pdf]

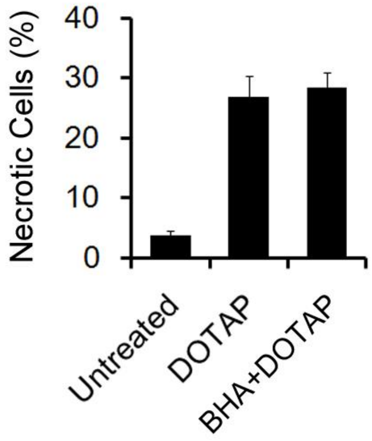

Supplementary information, Figure S4 No reduction in cell necrosis after the pretreatment of butylated hydroxyanisole (BHA).

Cell were pretreated with BHA (100μM) for 30 min, and then incubated with cationic DOTAP liposomes (25μg/ml) for 10 min. Cell necrosis were detected by flow cytometry with PI-staining. Data are mean±SEM; *n*=3.
