# Supplementary material for: Cationic nanocarriers induce cell necrosis through impairment of Na+/K+-ATPase and cause subsequent inflammatory response
Source: Cell Res. 2015 Jan 23;25(2):237–53. doi: 10.1038/cr.2015.9 (PMC4650577; doi:10.1038/cr.2015.9)
Supplement: Supplementary information, Figure S5 — No inhibitory effect of ouabain in TSQ-induced cell necroptosis. [file cr20159x5.pdf]

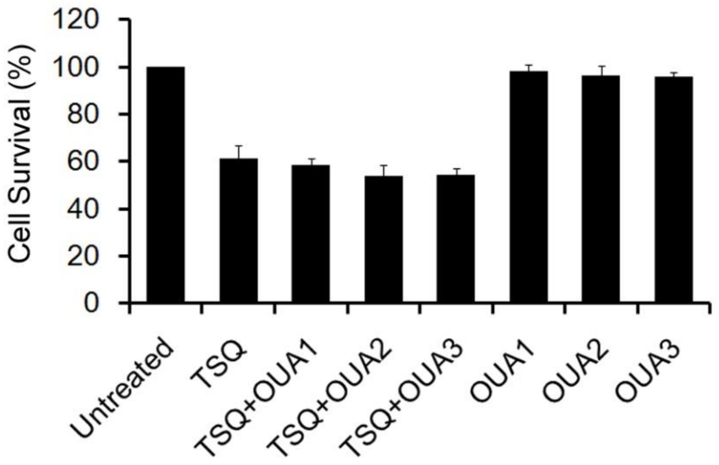

Supplementary information, Figure S5 No inhibitory effect of ouabain in TSQ-induced cell necroptosis.

Mouse dermal fibroblasts (MDFs) were isolated and pretreated with three concentration of ouabain (OUA1:1μM, OUA2: 2μM, OUA3: 5μM) for 30 min, then treated with hTNF (T, 100ng/ml), Smac-mimetic (S, 500nM) and QVD-OPH (Q, 5μM) for 18 h. Cell viability was determined by MTT assay. Data are expressed as mean ± SEM; *n*=3.
