# Supplementary material for: Cationic nanocarriers induce cell necrosis through impairment of Na+/K+-ATPase and cause subsequent inflammatory response
Source: Cell Res. 2015 Jan 23;25(2):237–53. doi: 10.1038/cr.2015.9 (PMC4650577; doi:10.1038/cr.2015.9)
Supplement: Supplementary information, Figure S6 — Cell necrosis induced by cationic nanocarriers of equal molarity. [file cr20159x6.pdf]

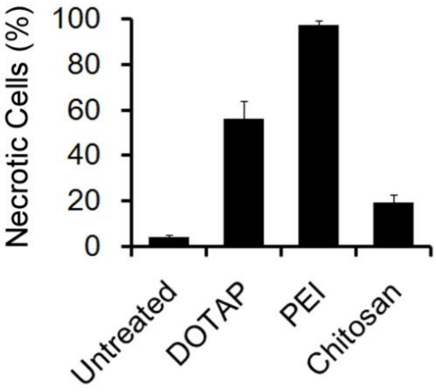

Supplementary information, Figure S6 Cell necrosis induced by cationic nanocarriers of equal molarity.

A549 cells were incubated with equal molarity (20mM) of DOTAP liposomes; PEI and Chitosan for 10 min and cell necrosis were detected by flow cytometry. Data are mean ± SEM; *n*=3.
