# Supplementary material for: Cationic nanocarriers induce cell necrosis through impairment of Na+/K+-ATPase and cause subsequent inflammatory response
Source: Cell Res. 2015 Jan 23;25(2):237–53. doi: 10.1038/cr.2015.9 (PMC4650577; doi:10.1038/cr.2015.9)
Supplement: Supplementary information, Figure S7 — The knockdown of ATPA1 and TRPM7 in A549 cell line. [file cr20159x7.pdf]

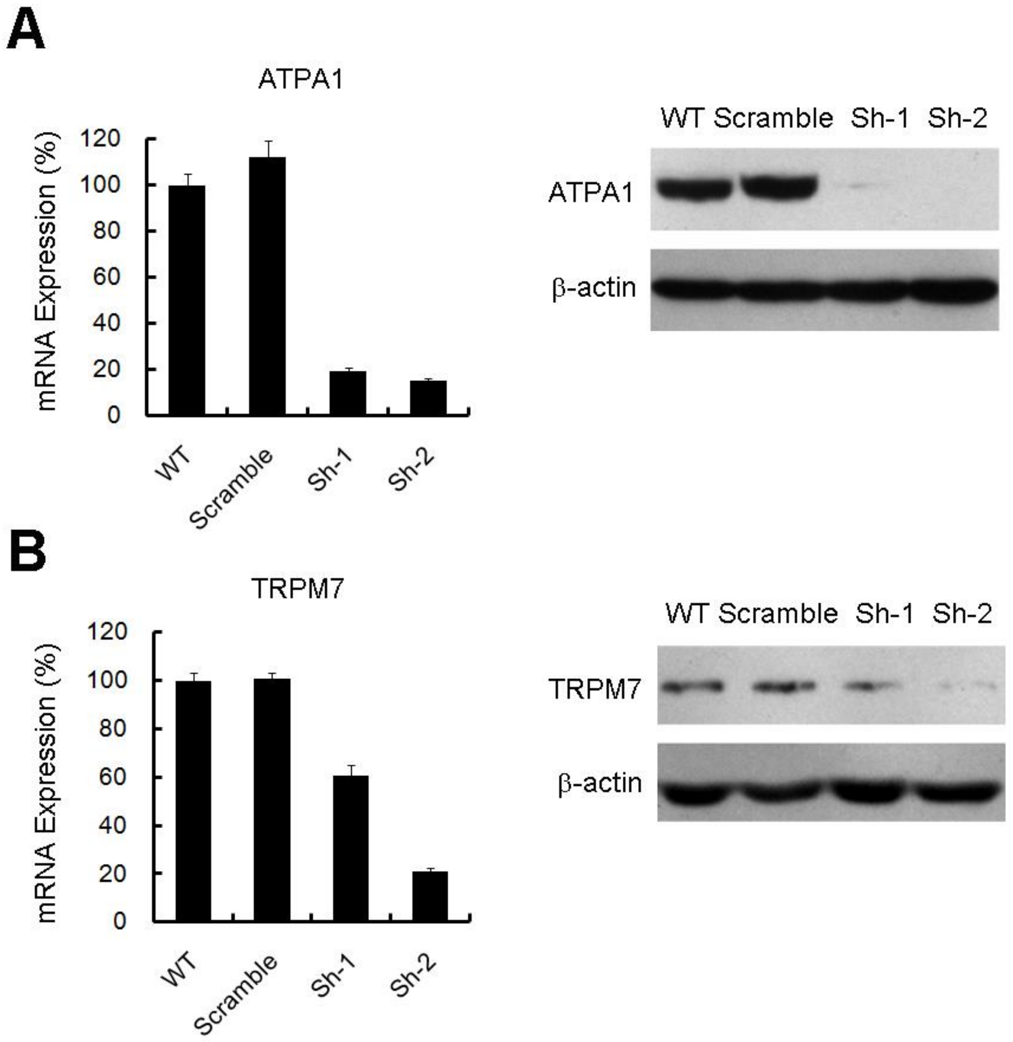

Supplementary information, Figure S7 The knockdown of ATPA1 and TRPM7 in A549 cell line. The knockdown of ATPA1 **(A)** and TRPM7 **(B)** in A549 cell line were confirmed by real-time PCR and western blotting. The Sh-2 group in both ATPA1- and TRPM7-knockdown cell line was further used in this experiment.
