# Supplementary material for: Cationic nanocarriers induce cell necrosis through impairment of Na+/K+-ATPase and cause subsequent inflammatory response
Source: Cell Res. 2015 Jan 23;25(2):237–53. doi: 10.1038/cr.2015.9 (PMC4650577; doi:10.1038/cr.2015.9)
Supplement: Supplementary information, Figure S8 — The reduction of pulmonary inflammation induced by mitochondria in Tlr9−/− mice. [file cr20159x8.pdf]

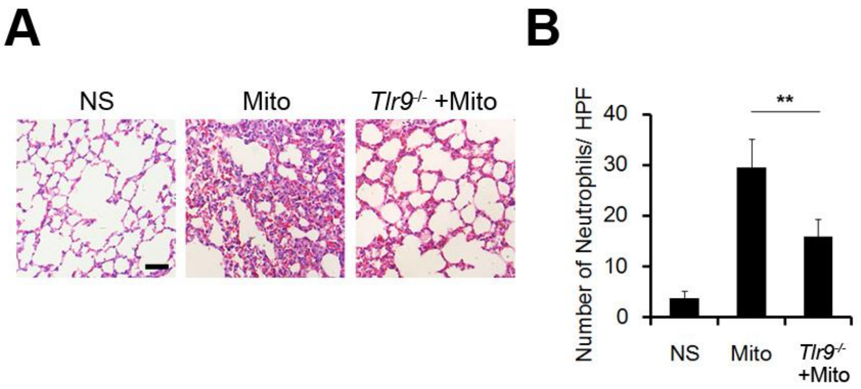

Supplementary information, Figure S8 The reduction of pulmonary inflammation induced by mitochondria in *Tlr9*$^{-/-}$ mice.

(A) Normal C57BL/6 mice and *Tlr9*$^{-/-}$ mice were treated with mitochondria (200μg /mouse) for 24 h and HE staining of lung sections was performed. Scale bar, 50 μm. **(B)** Esterase-stained neutrophils were counted in ten high power fields (HPFs). Data are mean ±SEM; n=3. $^{**}P$<0.01 compared with control group by Student's *t*-test.
